# A mechanism that ensures non-selective cytoplasm degradation by autophagy

**DOI:** 10.1038/s41467-023-41525-x

**Published:** 2023-09-19

**Authors:** Tetsuya Kotani, Yuji Sakai, Hiromi Kirisako, Chika Kakuta, Soichiro Kakuta, Yoshinori Ohsumi, Hitoshi Nakatogawa

**Affiliations:** 1 Cell Biology Center, Institute of Innovative Research, Tokyo Institute of Technology, Yokohama, 226-8501 Japan; 2 Department of Biosystems Science, Institute for Life and Medical Sciences, Kyoto University, Sakyo-ku, Kyoto 606-8507 Japan; 3 Laboratory of Morphology and Image Analysis, Biomedical Research Core Facilities, Juntendo University Graduate School of Medicine, Bunkyo-ku, Tokyo 113-8421 Japan; 4 School of Life Science and Technology, Tokyo Institute of Technology, Yokohama, 226-8501 Japan

**Keywords:** Macroautophagy, Membrane curvature, Autophagosomes

## Abstract

In autophagy, a membrane cisterna called the isolation membrane expands, bends, becomes spherical, and closes to sequester cytoplasmic constituents into the resulting double-membrane vesicle autophagosome for lysosomal/vacuolar degradation. Here, we discover a mechanism that allows the isolation membrane to expand with a large opening to ensure non-selective cytoplasm sequestration within the autophagosome. A sorting nexin complex that localizes to the opening edge of the isolation membrane plays a critical role in this process. Without the complex, the isolation membrane expands with a small opening that prevents the entry of particles larger than about 25 nm, including ribosomes and proteasomes, although autophagosomes of nearly normal size eventually form. This study sheds light on membrane morphogenesis during autophagosome formation and selectivity in autophagic degradation.

## Abstract (web summary)

How membrane morphology is regulated during autophagosome formation remains elusive. Here, authors reveal a mechanism by which the forming autophagosomal membrane expands with a large opening for non-selective sequestration of the cytoplasm.

## Introduction

Macroautophagy (hereafter autophagy) is a lysosome/vacuole-mediated degradation system for a cell’s own components^1,2^. Reflecting the diversity in degradation targets, which include proteins, nucleic acids, and organelles, autophagy has a wide variety of physiological functions, and its dysfunction is related to human diseases^3,4^. When autophagy is induced, a membrane cisterna called the isolation membrane (IM) (or phagophore) forms in the cytoplasm, expands, curves, and closes to form a spherical, double-membrane vesicle called the autophagosome. During this process, cellular components are selectively or non-selectively enclosed within autophagosomes, which subsequently fuse with lysosomes or vacuoles for degradation of the enclosed components. Previous studies have revealed the mechanisms by which the IM selectively takes in specific proteins or organelles^5,6^, and it is generally believed that cytoplasmic constituents are otherwise non-selectively sequestered as a natural consequence of autophagosome formation in the cytoplasm.

Theoretical studies suggested that the highly curved edge region of the IM is energetically unstable and therefore determines the morphology of the IM; a membrane cisterna like the IM cannot expand with the edge area beyond a critical point and therefore spontaneously bends and becomes spherical during its expansion^7,8^. Accordingly, proteins that bind to and stabilize the edge region can control the timing of IM bending and allow the membrane to expand with a larger opening. In this study, we found such proteins among autophagy-related (Atg) proteins in the budding yeast *Saccharomyces cerevisiae*.

## Results and discussion

### The Atg24 complex localizes to the opening edge of the IM

**Fig. 1:**
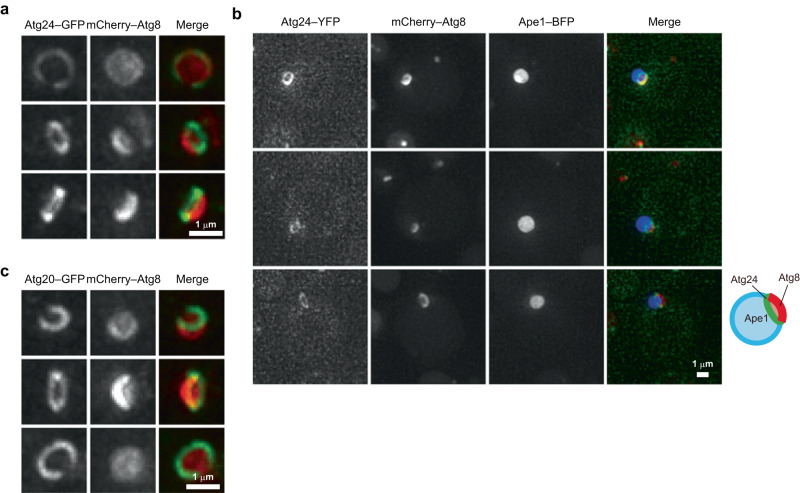
The Atg24 complex localizes to the opening edge of the IM. **a**–**c** Cells overexpressing Ape1 were treated with rapamycin for 4 h and analyzed by fluorescence microscopy. All images were subjected to maximum intensity projection. Scale bars, 1 μm. Experiments were repeated independently three (**a**, **c**) or two times (**b**) with similar results.

Atg24/Snx4 and Atg20/Snx42 are sorting nexins containing the phosphatidylinositol 3-phosphate (PI3P)-binding Phox homology (PX) domain and the membrane curvature-sensing/generating/stabilizing Bin/amphiphysin/Rvs (BAR) domain and form a heterodimeric complex^9^. The Atg24–Atg20 complex localizes to the site of autophagosome formation via PI3P binding^10^; however, its localization on expanding IMs has not been investigated. An experimental system has previously been established for observing cup-shaped IMs in yeast cells^11^. When the selective autophagic cargo aminopeptidase I (Ape1) is overexpressed, it forms giant liquid droplets in the cytoplasm^12^. When autophagy is induced in these cells, cup-shaped IMs expanding along the droplet surface can be visualized by fluorescence microscopy using the marker protein Atg8. We improved this system by integrating the *APE1* gene driven by the *GPD* promoter into a yeast chromosome and successfully observed IMs with a much higher frequency (Supplementary Fig. 1a, b). Using this system, we found that Atg24–GFP or Atg24–YFP, expressed by its own promoter from the original chromosomal locus, localized to the opening edge of the IM in a ring-like shape (Fig. 1a, b and Supplementary Video 1). Atg20–GFP or Atg20–YFP showed a similar localization pattern (Fig. 1c, Supplementary Fig. 1c and Supplementary Movie 1). Another sorting nexin, Snx41, which forms a complex with Atg24 competitively with Atg20^9^, also localized to the opening edge of the IM (Supplementary Fig. 1d). These results suggest that the Atg24–Atg20/Snx41 complex (hereafter the Atg24 complex) localizes to this specific part of the IM. Meanwhile, Vps17, an autophagy-unrelated sorting nexin (retromer subunit)^13^, localized to endosomes but not to the IM (Supplementary Fig. 1e).

### The Atg24 complex is required for autophagy of large particles

**Fig. 2:**
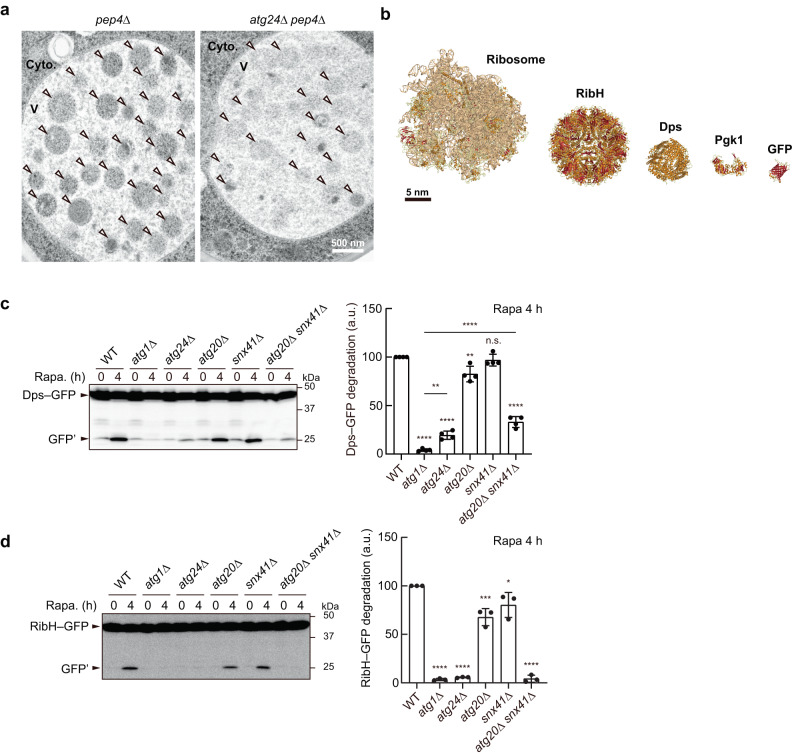
The Atg24 complex is required for the sequestration of large particles within the autophagosome. **a** Vacuolar protease-deficient cells were treated with rapamycin for 16 h. Autophagic bodies accumulated in the vacuole were observed by electron microscopy. Cyto. cytoplasm. V, vacuole. Arrowheads, autophagic bodies. Scale bar, 500 nm. Experiments were repeated independently three times with similar results. **b** Structures of the ribosome (PDBID: 4V7R), RibH (PDBID: 5MPP), Dps (PDBID: 1QGH), Pgk1 (PDBID: 1FW8), and GFP (PDBID: 4KW4). Scale bar, 5 nm. Yeast cells expressing Dps–GFP (**c**) or RibH–GFP (**d**) were treated with rapamycin (Rapa), and analyzed by immunoblotting using anti-GFP antibodies. WT wild-type. GFP’ GFP fragments generated by degradation of Dps–GFP or RibH–GFP in the vacuole. Bars represent means ± s.d. (*n* = 4 (**c**), 3 (**d**)) of ratio GFP′/(Dps–GFP or RibH–GFP + GFP′). *P < 0.05, **P < 0.01, ***P < 0.001, ****P < 0.0001, n.s. not significant (Tukey’s multiple comparisons test, three or four independent experiments). Source data and the exact adjusted *P*-values are provided as a Source Data file.

Previous studies have shown that degradation of GFP-fused Pgk1 (phosphoglycerate kinase), which is non-selectively sequestered within the autophagosome as part of the cytoplasm, occurs normally in cells deficient for the Atg24 complex^14^. In addition, vacuolar transport of cytoplasmically expressed alkaline phosphatase (ALP), another indicator of non-selective cytoplasm degradation by autophagy^15^, was normal in the absence of the Atg24 complex^10,16^. By contrast, the complex was reported to be important for autophagic degradation of peroxisomes, mitochondria, ribosomes, proteasomes, and fatty acid synthase as well as the autophagic transport of vacuolar enzymes^10,14,17,18^. All of these targets were suggested to be sequestered within autophagosomes in a selective manner. Thus, it has been thought that the Atg24 complex is specifically required for selective autophagy. We confirmed that the knockout of *ATG24* or *ATG20* decreased degradation of ribosomes (the large subunit protein Rpl25 fused to GFP) severely or partially, respectively, but not that of Pgk1–GFP in our experimental conditions (Supplementary Fig. 2a, b). We also confirmed that autophagic bodies, which are inner autophagosomal vesicles released into the vacuolar lumen upon outer autophagosomal membrane-vacuole fusion and accumulated in the vacuole of vacuolar protease-deficient cells^19^, in *ATG24* knockout (*atg24*Δ) cells contain Pgk1–GFP as well as those in wild-type cells (Supplementary Movie 2). While deletion of *SNX41* had little effect on ribosome degradation, double deletion of *ATG20* and *SNX41* caused severe defects (Supplementary Fig. 2a). In addition, we performed electron microscopy to directly observe ribosomes enclosed within autophagic bodies (Fig. 2a and Supplementary Fig. 2c). In *atg24*Δ cells, ribosomes (observed as electron-dense, black dots) were present in the cytoplasm as much as in wild-type cells, but autophagic bodies were strikingly absent from ribosomes, whereas autophagic bodies in wild-type cells contained ribosomes with a similar density to that in the cytoplasm. Thus, ribosomes are strictly excluded from autophagic sequestration in *atg24*Δ cells, despite a high abundance in the cytoplasm.

Given our finding that the Atg24 complex localizes to the opening edge of the IM and the fact that the complex contains the BAR domain, which can stabilize the edge region^20^, we hypothesized that the Atg24 complex acts to allow the IM to expand with a large opening and thereby enables non-selective cytoplasm sequestration, as opposed to the previous idea that the complex is specifically involved in selective autophagy. The sizes of Pgk1–GFP and ALP, which are degraded independently of the Atg24 complex, were about 10 nm (Fig. 2b). By contrast, ribosomes, proteasomes, and fatty acid synthase, whose degradation requires the Atg24 complex, were all about 30 nm. We supposed that the IM develops with a small opening in *atg24*Δ cells and therefore excludes large complexes. To examine this possibility, we employed two bacterial proteins as model particles: the ferritin-like, DNA-binding protein Dps from *Listeria innocua*, which forms a tetrahedral dodecamer of about 9 nm^21^, and the lumazine synthase RibH from *Aquifex aeolicus*, which forms a spherical hexacontamer of about 15 nm^22^ (Fig. 2b). These proteins were fused with GFP (about 5 nm) and expressed in yeast cells. The particle sizes of the Dps–GFP dodecamer and the RibH–GFP hexacontamer were expected to be about 20 and 25 nm, respectively. Both of the fusion proteins were evenly dispersed throughout the cytoplasm (Supplementary Movie 3). We also showed that Dps–GFP was not sedimented by ultracentrifugation that sedimented ribosomes, confirming that this fusion protein does not form particles larger than ribosomes (Supplementary Fig. 2f). RibH–GFP was sedimented by ultracentrifugation but not by centrifugation that partially sedimented ribosomes, suggesting that this model particle is larger than the Dps–GFP particle and a little smaller than ribosomes (Supplementary Fig. 2f). Autophagic degradation of GFP-fused proteins generates vacuolar protease-resistant GFP fragments^23^. When yeast cells expressing Dps–GFP or RibH–GFP were treated with the Tor kinase complex 1 (TORC1) inhibitor rapamycin, which strongly induces non-selective cytoplasm degradation by autophagy^24^, GFP fragments appeared for both Dps–GFP and RibH–GFP in wild-type cells but not in autophagy-deficient *atg1*Δ cells, suggesting that these fusion proteins are degraded by autophagy (Fig. 2c, d). Degradation of Dps–GFP was less efficient in *atg24*Δ and *atg20*Δ *snx41*Δ cells than in wild-type cells, and that of RibH–GFP was almost completely abolished by deletion of *ATG24* or double deletion of *ATG20* and *SNX41*. These results suggest that the IM prevents the entry of cytoplasmic components larger than about 25 nm in the absence of the Atg24 complex. We also examined autophagic degradation of Dps–GFP and ribosomes in cells with different levels of Atg24 (Supplementary Fig. 2g, h). The level of Atg24 (Atg24–FLAG) decreased when the promoter was replaced with the *ATG14* promoter and further decreased when expressed with the *DDI2* promoter. Cells expressing Atg24 with the *ATG14* promoter was defective in ribosomal degradation but normal for Dps–GFP degradation. Meanwhile, degradation of both the ribosome and Dps–GFP was defective in cells expressing Atg24 from the *DDI2* promoter. These results suggest that the size of the opening of the IM correlates with cellular levels of the Atg24 complex.

### The Atg24 complex acts to expand the IM opening

**Fig. 3:**
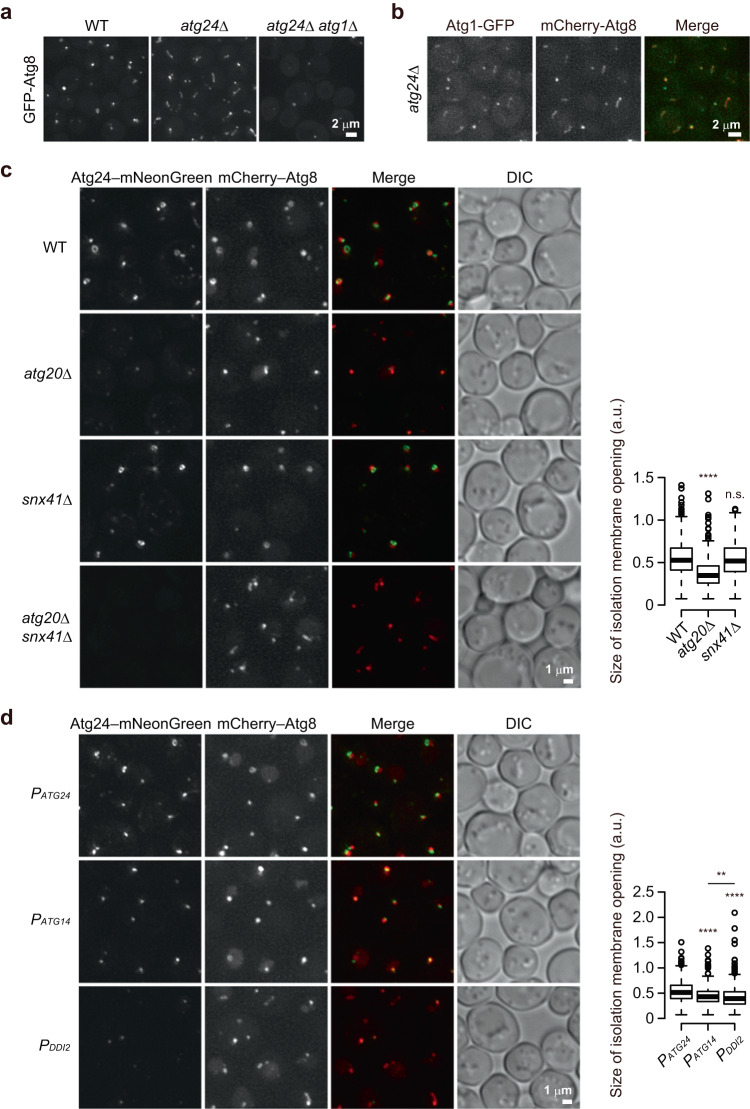
The Atg24 complex expands the opening of the IM. **a** Yeast cells expressing GFP–Atg8 were treated with rapamycin for 1 h and observed under a fluorescence microscope. Scale bar, 2 μm. All images were subjected to maximum intensity projection. Experiments were repeated independently two times with similar results. **b** Yeast cells expressing mCherry–Atg8 and Atg1–GFP were treated with rapamycin for 1 h and observed under a fluorescence microscope. Scale bar, 2 μm. All images were subjected to maximum intensity projection. Experiments were repeated independently two times with similar results. **c**, **d** Cells grown to mid-log phase were treated with rapamycin for 2 h and observed under a fluorescence microscope. Atg24–mNeonGreen was expressed by the *ATG24* (*P*_*ATG24*_), *ATG14* (*P*_*ATG14*_), or *DDI2* (*P*_*DDI2*_) promoter, respectively (**d**). DIC, Differential interference contrast microscopy. Scale bar, 1 μm. All images except for the DIC image were subjected to maximum intensity projection. The sizes of IM openings were measured as described in Methods and are shown as boxplots. The center line of box represents the median. The bottom and top ends of the box represent the first and third quartiles, respectively. The edge of the upper whisker represents the largest point less than 1.5 times the height of the box plus the third quartile. The edge of the lower whisker represents the smallest point above the first quartile minus 1.5 times the height of the box. **P < 0.01; ****P < 0.0001; n.s. not significant (Dunn’s multiple comparisons test, two independent experiments). (*n* = 487 (**c** WT), 483 (**c** *atg20*Δ), 483 (**c** *snx41*Δ), 601 (**d** *P*_*ATG24*_), 561 (**d** *P*_*ATG14*_), 481 (**d** *P*_*DDI2*_)). n number of IMs examined over two independent experiments. Source data and the exact adjusted *P*-values are provided as a Source Data file.

Next, we tried to measure the size of the opening of IMs formed with or without the Atg24 complex by electron microscopy. In wild-type cells (without Ape1 overexpression), we observed two autophagosome-related structures in the cytoplasm: IMs with a clear opening and closed ring-like structures containing ribosomes, which should represent the cross-section of complete autophagosomes or that of IMs out of the opening (Supplementary Fig. 2i). Meanwhile, in *atg24*Δ cells, we observed closed ring-like and elliptical structures, both of which scarcely contained ribosomes, but could not find IMs with a clear opening. We also performed serial section transmission electron microscopy to capture the opening of IMs in *atg24*Δ cells. During this analysis, we found that IMs in *atg24*Δ cells are tubular and waving, while they are spheroidal in wild-type cells. (Supplementary Fig. 2j). Fluorescence microscopy also revealed that *atg24*Δ cells formed tubular structures positive for Atg8 in a manner dependent on Atg1, which is responsible for autophagosome formation, and Atg1 also localizes to these structures as with IMs formed in wild-type cells^11^, suggesting that IMs expand into a tubular shape in the absence of Atg24. (Fig. 3a, b). This morphological change in the IM precluded us from measuring the size of its opening in electron microscopy sections; we could not distinguish whether a membrane-less area in the IM represented its opening or curved part, which was just invisible because of diagonal cutting. Thus, we estimated the size of the IM opening by fluorescent microscopy. Since the rim of the IM can be visualized as a ring or punctum (small ring) of Atg24, we measured the size of rings and puncta of Atg24–mNeonGreen associated with the IM labeled with mCherry–Atg8 in the absence of giant Ape1 droplets. Compared to wild-type cells, Atg24–mNeonGreen formed smaller rings and puncta in *atg20*Δ cells, whereas the size of Atg24–mNeonGreen rings and puncta in *snx41*Δ cells was almost the same as that in wild-type cells (Fig. 3c). (The size of the IM opening could not be estimated in *atg20*Δ *snx41*Δ cells, because Atg24–mNeonGreen did not form rings/puncta in these cells.) In addition, the size of Atg24–mNeonGreen rings/puncta decreased as the level of Atg24–mNeonGreen was lowered by promoter replacement (Fig. 3d). These results suggest that the IM opening is indeed smaller in cells defective in the Atg24 complex than in wild-type cells.

By contrast, the size and number of autophagic bodies did not differ largely between wild-type and *atg24*Δ cells (Supplementary Fig. 2d, e), providing important insight into the mechanism underlying autophagosome biogenesis; the final size of the autophagosome and the overall efficiency of autophagosome formation can be determined independently of the opening size of the expanding IM. However, in the fission yeast *Schizosaccharomyces pombe*, cells lacking Atg20 and Atg24 homologs accumulated autophagosomes smaller than those of control cells^25^. Therefore, multiple mechanisms may govern autophagosome morphogenesis, and these mechanisms may vary among organisms.

### The Atg24 complex is required for IM expansion on giant Ape1 droplets

**Fig. 4:**
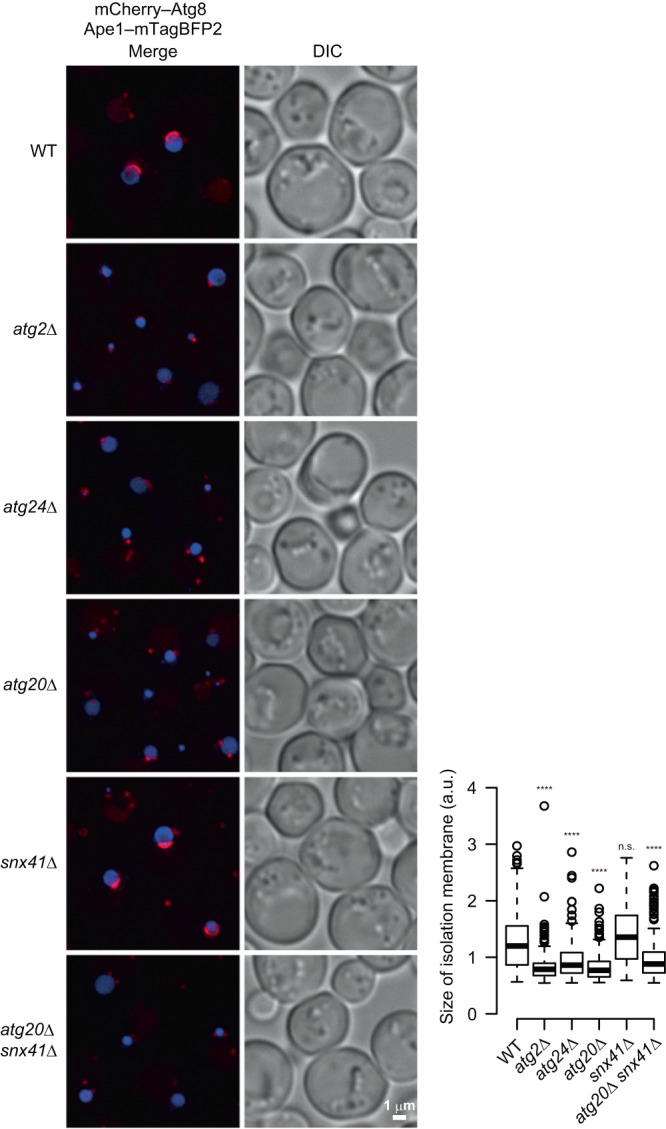
The Atg24 complex is important for the expansion of the IM along the surface of giant Ape1 droplets. Cells overexpressing Ape1 were treated with rapamycin for 4 h and IMs labeled with mCherry–Atg8 were observed by fluorescence microscopy. Scale bar, 1 μm. All images except for the DIC image were subjected to maximum intensity projection. The graph shows the sizes of IMs, measured as described in Methods and shown as boxplots as defined in Fig. 3c. ****P < 0.0001; n.s. not significant (Dunn’s multiple comparisons test, two independent experiments). (*n* = 220 (WT), 220 (*atg2*Δ), 245 (*atg24*Δ), 251 (*atg20*Δ), 196 (*snx41*Δ), 261 (*atg20*Δ *snx41*Δ)). n number of IMs examined over two independent experiments. Source data and the exact adjusted *P*-values are provided as a Source Data file.

We also found that the Atg24 complex is important for the expansion of the IM along the surface of giant Ape1 droplets (Fig. 4 and Supplementary Fig. 3a–c, e, f), although the complex is dispensable for the autophagic sequestration of Ape1 droplets of normal size in cells treated with rapamycin (Supplementary Fig. 3d)^10^. IM expansion on giant Ape1 droplets was severely impaired by the knockout of *ATG24* or *ATG20* or the double knockout of *ATG20* and *SNX41*, whereas the membrane normally expanded in *SNX41* single knockout cells (Fig. 4 and Supplementary Fig. 3a–c). In addition, lowering the protein levels of Atg24 also reduced the size of IMs formed on giant Ape1 droplets (Supplementary Fig. 3e, f). These results are consistent with the notion that the Atg24 complex is required for the IM to expand with a large opening (the opening of the IM needs to enlarge during its expansion along the surface of giant Ape1 droplets). Although *ATG20* disruption only partially decreased degradation of ribosomes, Dps, and RibH (Fig. 2c, d and Supplementary Fig. 2a), it abolished IM expansion on giant Ape1 droplets as severely as disruption of *ATG24* or *ATG2*, which encodes a lipid transfer protein essential for autophagosome formation^26–28^. The requirement of Atg20 may increase in the sequestration of larger cargoes such as giant Ape1 droplets into the autophagosome. Alternatively, since Atg20 was reported to interact with Atg11, which is involved in the initiation of autophagosome formation on selective cargoes^16,29,30^, the more severe effect of *ATG20* knockout on IM expansion on giant Ape1 droplets may be attributed to the loss of this specific function of Atg20. In addition, we found that the fluorescence intensity of Atg24–GFP rings/puncta associated with IMs was largely decreased by deletion of *ATG20*, suggesting that this decrease in the localization of Atg24 to the IM opening may also contribute to defects in IM expansion on giant Ape1 droplets in the absence of Atg20 (Supplementary Fig. 3a). We also showed that the localization of Atg20 and Snx41 to the IM opening depended on Atg24 (Supplementary Fig. 3b, c).

### Significance of the PX and BAR domains of the Atg24 complex

**Fig. 5:**
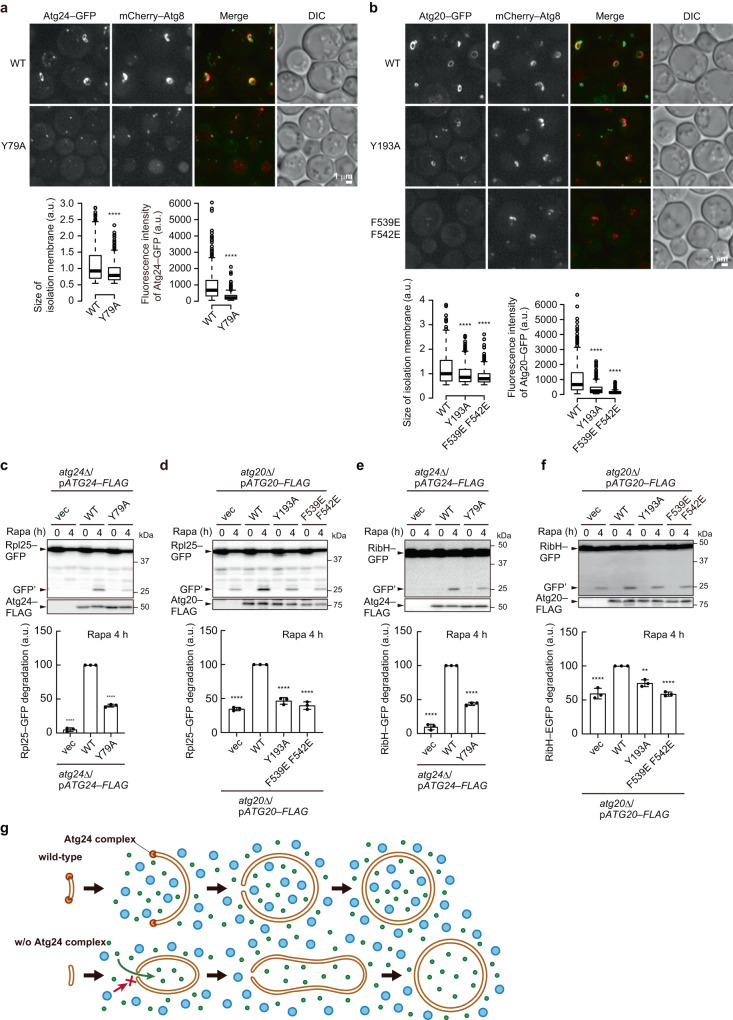
Analyses of PX and BAR domain mutants of Atg24 and Atg20. Atg24–GFP (**a**) or Atg20–GFP (**b**) cells overexpressing Ape1 were treated with rapamycin for 4 h and observed under a fluorescence microscope. Scale bar, 1 μm. All images except for the DIC image were subjected to maximum intensity projection. The graphs show the sizes of IMs and fluorescence intensities of Atg24–GFP and Atg20–GFP associated with IMs, measured as described in Methods and shown as boxplots as defined in Fig. 3c. ****P < 0.0001 (Two-tailed Mann–Whitney test (**a**) and Dunn’s multiple comparisons test (**b**), two independent experiments). (*n* = 429 (**a** WT), 434 (**a** Y79A), 468 (**b** WT), 453 (**b** Y193A), 421 (**b** F539E F542E)). n number of IMs examined over two independent experiments. **c**–**f** Atg24 or Atg20 mutant cells expressing Rpl25–GFP (**c**, **d**) or RibH–GFP (**e**, **f**) were treated with rapamycin and examined by immunoblotting. Graphs show mean ± s.d. (*n* = 3) of ratio GFP′/(Rpl25–GFP or RibH–GFP + GFP′). **P < 0.01; ****P < 0.0001 (Tukey’s multiple comparisons test, three independent experiments). **g** Model of the function of the Atg24 complex. In wild-type cells, the Atg24 complex stabilizes the edge of the IM and thereby allows the IM to expand with an opening large enough for non-selective cytoplasm engulfment. In the absence of the Atg24 complex, the IM expands with a small opening that excludes large particles, such as ribosomes and proteasomes, resulting in a difference in the concentration of these particles between the IM interior and the cytoplasm. This concentration difference generates osmotic pressure across the membrane, which tubulates the IM. Source data and the exact (adjusted) *P*-values are provided as a Source Data file.

We showed that the Y79A or Y193A mutation in the PX domain of Atg24 or Atg20, respectively^10^, and the F539E F542E mutation in the BAR domain of Atg20^16^ impaired the localization of these proteins to the IM edge, and the IM labeled with mCherry–Atg8 expanded less efficiently on giant Ape1 droplets in these mutant cells (Fig. 5a, b). These mutations also reduced autophagic degradation of ribosomes and RibH–GFP but not that of Pgk1–GFP (Fig. 5c–f and Supplementary Fig. 3g, h). These results suggest that the Atg24 complex localizes to the edge of the IM opening by binding to PI3P in the PX domains and sensing the high membrane curvature in the BAR domains.

BAR domain binding is likely to stabilize the edge region and thereby retard the timing of IM bending during its expansion (Fig. 5g). Consequently, the IM develops with an opening large enough to take in cytoplasmic materials in a non-selective manner. Without edge stabilization by the Atg24 complex, the IM expands with a small opening that excludes large molecular complexes such as ribosomes, proteasomes, and fatty acid synthase, although autophagosomes of nearly normal size can form eventually. Importantly, this model is not contradictory to previous reports that ribosomes, proteasomes, and fatty acid synthase are selectively sequestered within autophagosomes^18,31^. The ability of the Atg24 complex to enlarge the IM opening would also be important in selective autophagy of these targets.

### Mathematical analysis on IM tubulation in Atg24 complex-deficient cells

In *atg24*Δ cells, the IM with a small opening expands into a tubular shape (Fig. 3a, b and Supplementary Fig. 2j). In these cells, large particles including ribosomes do not enter the IM through the opening, causing a difference in the concentration of these particles between the IM interior and the cytoplasm, which could generate osmotic pressure across the membrane and thereby affect the morphology of the IM. To verify this hypothesis, we conducted a mathematical model analysis, in which membrane shapes were determined based on the elastic bending energy model^32^ (see Methods for details). Given our experimental results that the IM does not allow the entry of cytoplasmic components larger than about 25 nm in *atg24*Δ cells, the size of the IM opening was set to 25 nm. The total membrane area was fixed to π μm^2^, which corresponded to that of an autophagosome of 0.7 μm in diameter. The result of analysis suggested that osmotic pressure generated by a concentration difference of a few μM causes the tubulation of the IM (Supplementary Fig. 4b). Given the estimation that the intracellular ribosome concentration is ~10 μM^22,33^, osmotic pressure generated by the total concentration difference of large particles that cannot enter the IM is likely to be sufficient to tubulate the IM in *atg24*Δ cells. Given our observation that autophagic bodies in *atg24*Δ cells are spherical as well as those in wild-type cells, we performed time-lapse imaging to examine whether tubular IMs finally become spherical autophagosomes in *atg24*Δ cells. While IMs visualized as puncta of GFP-Atg8 increased in size and often expanded into a ring-like shape in wild-type cells (Supplementary Movie 4), most of the IMs that expanded into a tubular shape finally contracted into punctate structures in the cytoplasm in *atg24*Δ cells (Supplementary Movie 5). These results suggest that spherical autophagosomes form even in the absence of the Atg24 complex. What drives this tubule-to-sphere transition in IM morphology in *atg24*Δ cells remains to be clarified.

### *ATG24* knockout decreases cell viability under nitrogen starvation

**Fig. 6:**
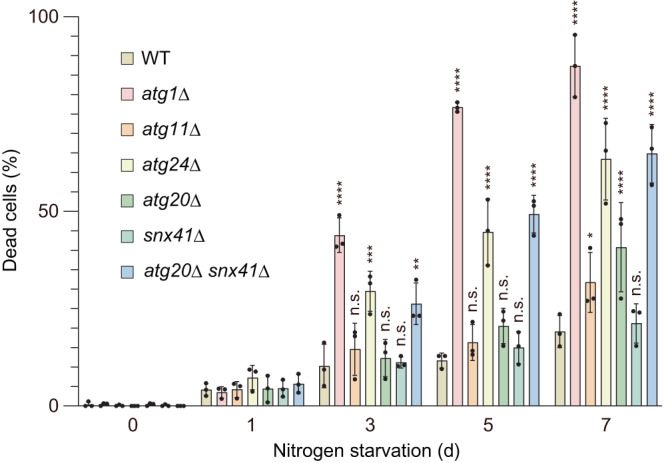
*ATG24* knockout increases cell sensitivity to nitrogen starvation. Yeast cells were incubated in nitrogen-starvation medium for 7 days and dead cells were stained with phloxine B. At least 100 cells were examined for each strain and the percentage of cells stained with phloxine B was determined. Bars represent means ± s.d. (*n* = 3) of ratio of phloxine B positive cells. *P < 0.05, **P < 0. 01, ***P < 0.001, ****P < 0.0001, n.s. not significant (Tukey’s multiple comparisons test (2way ANOVA), three independent experiments). Source data and the exact adjusted *P*-values are provided as a Source Data file.

Finally, we investigated the physiological impact of the Atg24 complex-mediated autophagic degradation of large molecular complexes. As previously reported, yeast cells defective in all macroautophagy-related pathways, such as *atg1*Δ cells, exhibit reduced viability during nitrogen starvation (Fig. 6)^34^. We found that the knockout of *ATG24* and double knockout of *ATG20* and *SNX41* also largely promoted cell death under nitrogen starvation conditions. In agreement with the extent of defects in degradation of large cytoplasmic particles, *atg20*Δ cells and *snx41*Δ cells showed milder and no defects in viability under nitrogen starvation, respectively. We also showed that knockout of *ATG11*, which impairs selective autophagy of organelles, including mitochondria, peroxisomes, the endoplasmic reticulum, and the nucleus, but not ribosomes, proteasomes, and fatty acid synthase, only slightly affected cell viability under nitrogen starvation. These results suggest that degradation of large cytoplasmic complexes is beneficial for yeast viability during nitrogen starvation.

In this study, we discovered a mechanism required for the IM to expand with a large opening and thus for non-selective cytoplasm sequestration and degradation in autophagy. Our findings could also explain the requirement for the Atg24 complex in selective autophagy of organelles, in which large organelle fragments (e.g., fragmented mitochondria of about 200–300 nm in selective autophagy of mitochondria^35,36^) are enclosed within autophagosomes. Without the Atg24 complex, IMs tightly associated with large, rigid cargoes cannot expand in selective autophagy of those cargoes as the IM opening needs to enlarge during its expansion along the surface of those cargoes, whereas in non-selective autophagy, the IM bends to reduce the highly-curved, unstable edge area and expands with a small opening that does not allow the entry of large particles diffusing in the cytoplasm. Mammals have >30 sorting nexins^37^, some of which may play similar roles in selective and/or non-selective autophagy in the cells. In addition, other membrane edge-stabilizing proteins could also affect the morphology of the IM in a similar manner, and such a role has recently been proposed for ATG2^8^. This study provides a new direction for research on the still unresolved, important issues in autophagy: how membrane morphogenesis and selectivity in degradation are controlled.

## Methods

### Yeast strains and media

The *S. cerevisiae* strains used in this study were derived from W303-1A^38^ and are listed in Table S1. Gene deletion and fluorescent protein tagging were performed using a PCR-based method^39^. pRS303- and pRS306-based plasmids^40^ were linearized with the appropriate restriction enzymes prior to introduction into yeast cells. The cells were cultured at 30 °C in YPD medium (1% yeast extract, 2% peptone, and 2% glucose) or in SD + CA + ATU medium (0.17% yeast nitrogen base without amino acids and ammonium sulfate [YNB w/o aa and as], 0.5% ammonium sulfate, 0.5% casamino acids, and 2% glucose supplemented with 0.002% adenine sulfate, 0.002% tryptophan, and 0.002% uracil). When cells carried plasmids derived from pRS316, SD + CA + AT (SD + CA + ATU without uracil) was used. To induce autophagy, cells were treated with 200 ng/ml rapamycin or incubated in SD-N nitrogen starvation medium (0.17% YNB w/o aa and as and 2% glucose) or in SGly+CA + ATU medium (0.17% YNB w/o aa and as, 0.5% ammonium sulfate, 0.5% casamino acids, and 2% glycerol supplemented with 0.002% adenine sulfate, 0.002% tryptophan, and 0.002% uracil).

### Plasmids

The plasmids used in this study are listed in Table S2. pRS306-based plasmids for the expression of Atg20–EGFP and Atg24–EGFP were constructed as follows. The coding sequences of Atg20–EGFP and Atg24–EGFP with their original promoters (1000 bp upstream of the start codon) and the *PGK1* terminator (250 bp downstream of the stop codon) were amplified by PCR from genomic DNA from a yeast strain in which genomic *ATG20* and *ATG24* were tagged with EGFP. The resultant PCR products were cloned into pRS306 using the Gibson Assembly Kit (New England Biolabs, Ipswich, MA). pRS316-based plasmids for the expression of Atg20–FLAG and Atg24–FLAG were constructed as follows. DNA fragments excised from pRS306–*ATG20*–*EGFP* and pRS306–*ATG24*–*EGFP* using SacI and Sal1 were cloned into the SacI and XhoI sites on the pRS316 vector to construct pRS316–*ATG20*–*EGFP* and pRS316–*ATG24*–*EGFP*. The GFP moiety of these plasmids was exchanged with the 3×FLAG sequence using a PCR-based method to construct pRS316–*ATG20*–*FLAG* and pRS316–*ATG24*–*FLAG*. Point mutations were introduced into these plasmids using the PrimeSTAR Mutagenesis Basal Kit (TaKaRa, Kusatsu, Japan).

pRS303-based plasmids for the expression of Dps–EGFP and RibH–EGFP were constructed as follows. DNA fragments of the codon-optimized coding sequences of Dps and RibH created by gene synthesis (GenScript, Piscataway, NJ), the coding sequence of EGFP with the *PGK1* terminator, and the *CYC1* promoter derived from pYM-N11^39^ were cloned into the pRS303 vector.

pRS303- and pRS306-based plasmids for the overexpression of Ape1 were constructed as follows. The coding sequence for Ape1 with the *GPD* promoter was amplified by PCR from genomic DNA from a yeast strain in which the promoter of the *APE1* gene was exchanged with the *GPD* promoter. The resultant PCR products were cloned into pRS303 using the Gibson Assembly Kit. DNA fragments excised from pRS303–*P*_*GPD*_*–APE1* using SacI and Sal1 were cloned into the SacI and XhoI sites on the pRS306 vector to construct pRS306–*P*_*GPD*_–*APE1*.

pRS303-based plasmids for the expression of Rpl25–EGFP was constructed as follows. DNA fragment excised from pRS316–*RPL25*–*EGFP* using SalI and Not1 was cloned into the same sites on the pRS303 vector to construct pRS303–*RPL25*–*EGFP*.

### Fluorescence microscopy

Fluorescence microscopy of the IM was performed using a DeltaVision Elite microscope system (GE Healthcare, Chicago, IL) equipped with a CMOS camera (pco.edge 5.5; PCO AG), a 100× objective lens (UPlanSApo 100×/1.40 oil; Olympus, Tokyo, Japan). Twenty-five (Supplementary Fig.4a) or thirty (other figures) z-stack images at 0.2-μm intervals were obtained and deconvolved using SoftWoRx 7.0.0 (GE Healthcare). Maximum intensity projection was performed using Fiji (ImageJ Version 1.53t)^41,42^. The perimeter of the IM was measured using the ImageJ Script described in ImageJ Script S1 (Fig. 4), ImageJ Script S2 (Fig. 5a, b and Supplementary Fig. 3a–c, e, f). Half of the perimeter was used as the size of the IM. The fluorescence intensity of Atg24/Atg20/Snx41–GFP associated with the IM was measured using the regions of interest (ROI) used to measure the size of the IM. To measure the opening of the IM, rings and puncta of Atg24–mNeonGreen were extracted using the ImageJ Script described in ImageJ Script S3 (Fig. 3c, d). The major axis of the rings/puncta was used as the size of the IM opening. When signals clearly different from IMs were extracted, they were manually excluded.

Fluorescence microscopy to observe Dps–GFP and RibH–GFP was performed using an inverted fluorescence microscope (IX83; Olympus) equipped with a 150× objective lens (UAPON 150XOTIRF, NA/1.45; Olympus) and an electron-multiplying CCD camera (ImagEM C9100-13; Hamamatsu Photonics). A 488-nm blue laser (50 mW; Coherent) was used for excitation of GFP. Fluorescence was filtered with a dichroic mirror reflecting 405-nm, 488-nm, and 588-nm wavelengths (Olympus) and split into two channels using the DV2 multichannel imaging system (Photometrics) equipped with a Di02-R594-25 × 36 dichroic mirror (Semrock). Fluorescence was further filtered with a TRF59001-EM ET bandpass filter (Chroma) for the GFP channel. Images were acquired using MetaMorph Version 7.10.0.119 (Molecular Devices, San Jose, CA) and processed using Fiji.

### Immunoblotting

Yeast cells pellets were suspended in ND solution [0.2 M NaOH and 100 mM dithiothreitol (DTT)] and incubated on ice for 15 min. Cell suspensions were mixed with an equal volume of 20% trichloroacetic acid and incubated on ice for 15 min. The samples were centrifuged at 17,700 g for 5 min. The pellets were washed with ice-cold acetone, dried, and suspended in SDS sample buffer [100 mM Tris-HCl (pH 7.5), 2% SDS, 20 mM DTT, 10% glycerol, and a trace amount of bromophenol blue] by mixing at 65 °C for 10 min. After boiling for 3 min and centrifugation at 17,700 g for 1 min, the supernatants were analyzed by immunoblotting. For immunoblotting, antibodies against GFP (JL-8(632381); Clontech, Mountain View, CA; dilution 1:2000), FLAG (M2 (F1804); Sigma Aldrich, St. Louis, MO; dilution 1:2000), mTagBFP2 (AB233; Evrogen, Moscow, Russia; dilution 1:5000), Ape1 (anti-API-2;^43^; dilution 1:5000), Pgk1 (22C5D8; Invitrogen, Waltham, MA; dilution 1:10000), mouse lgG (HRP-conjugated) (315-035-003; Jackson lmmunoResearch, West Grove, PA; dilution 1:5000) and rabbit lgG (HRP-conjugated) (111-035-144; Jackson lmmunoResearch, West Grove, PA; dilution 1:5000) were used. Immunoblots were detected using ImageQuant LAS4000 Version 1.2 (GE Healthcare) or FUSION FX/EvolutionCapt Version 18.0.12.0 (Vilber; Eberhardzell, Germany).

### Cell fractionation

Yeast cells were grown to mid-log phase, converted to spheroplasts, and incubated in 0.5 × SD + CA + ATU medium containing 1 M sorbitol and 200 ng/ml rapamycin at 30 °C for 1 h. The spheroplasts were then suspended in Cell Fractionation buffer (20 mM HEPES-KOH [pH 7.2], 0.2 M sorbitol, 5 mM MgCl_2_, 150 mM NaCl, and 2 × Complete protease inhibitor cocktail [Roche]) and lysed by passing them through 3 µm-pore-sized polycarbonate membranes (Merck, Darmstadt, Germany). The lysates were clarified by centrifugation at 1000 g for 5 min, and the resulting supernatants were further centrifuged at 15,000 g for 30 min or at 100,000 g for 1 h to separate them into supernatant and pellet fractions. These samples were analyzed by immunoblotting with antibodies against GFP and Pgk1.

### Electron microscopy

Electron microscopy was performed by Tokai Electron Microscopy, Inc. based on a rapid freezing and freeze-fixation method. Yeast cells were sandwiched between copper disks and frozen in liquid propane at −175 °C. The frozen samples were placed in 2% osmium tetroxide in acetone and 2% distilled water (Fig. 2a and Supplementary Fig. 2c) or 2% glutaraldehyde and 2% tannic acid in ethanol (Supplementary Fig. 2i, j) at −80 °C. These samples were dehydrated, embedded in resin (Quetol-651; Nisshin EM Co., Tokyo, Japan) and polymerized. The polymerized resins were used to obtain ultra-thin sections with a diamond knife and an ultramicrotome (ULTRACUT UCT; Leica, Wetzlar, Germany), and the sections were placed on copper grids. They were stained with 2% uranyl acetate, rinsed with distilled water, and then stained with secondary lead stain solution (Sigma-Aldrich). Electron microscopy was performed using a transmission electron microscope (JEM-1400Plus; JEOL Ltd., Tokyo, Japan) at an acceleration voltage of 100 kV, and digital images were obtained using a CCD camera (EM-14830RUBY2; JEOL Ltd.). For serial section transmission electron microscopy (Supplementary Fig. 2j), serial ultrathin sections were cut with an ultramicrotome, mounted on glass coverslips, and stained as described above. These coverslips were coated with carbon using a carbon coater CADE-4T (Meiwa Fosis, Tokyo, Japan). Specimens were examined with a scanning electron microscope Helios NanoLab 660 (FEI, Oregon, USA). In Supplementary Fig. 2d, e, round membrane structures within the vacuole were analyzed as autophagic bodies. In Supplementary Fig. 2i, j, we identified round membrane structures containing cytoplasmic components (ribosomes) and those containing few ribosomes as autophagosome-related structures (autophagosomes and IMs) in wild-type and *atg24*Δ cells, respectively.

### Cell viability assay

Yeast cells grown to mid-log phase in YPD medium were incubated in SD-N nitrogen starvation medium for indicated time periods. Dead cells were stained with phloxine B (f.c. 5 μg/ml).

### Modeling of IM morphology in osmotic pressure differences

To investigate whether the osmotic pressure affects the morphology of the IM, we conducted a mathematical model analysis. Since morphological changes of the IM during autophagosome formation occur slowly, taking minutes, compared to the mechanical relaxation, which takes milliseconds, the morphology at each time is considered to be in equilibrium^8,44^. The membrane shapes at equilibrium were determined from the elastic bending energy model^32^. In the elastic bending energy model, the free energy $F$ with the osmotic pressure $P$ was given by

1 $$F=\mathop{\sum}\limits_{i=+,-}\int \frac{{\kappa }_{b}}{2}{J}_{i}^{2}d{A}_{i}-{PV}.$$

Here, the first term is the elastic bending energy with the total curvature, $${J}_{i}$$, of the inner ($$i=-$$) and outer ($$i=+ $$) membranes. The spontaneous curvature was assumed to be zero because the inner and outer membranes contain relatively few proteins^45^. The volume of the lumen of the isolated membrane is small, and the inner and outer membranes are considered to remain nearly parallel. Thus, the IM was assumed to have closely juxtaposed inner and outer membranes with the same curvature and area^8,44^. The bending modulus is $${\kappa }_{b}=20{k}_{B}T$$ with the Boltzmann constant $${k}_{B}$$ and temperature $$T$$. The second term is the osmotic pressure energy with the pressure difference $$P$$ and the volume $$V$$ covered by the IM, respectively. The rim is in principle controlled by a curvature-inducing protein that stabilizes the highly curved rim, and the energy is considered to be stable. For an axisymmetric membrane, the free energy was rewritten as

2 $$F=2\pi {\int }_{0}^{{s}_{1}}\left[{\kappa }_{b}x{\left(\dot{\theta }+\frac{\sin \theta }{x}\right)}^{2}-\frac{P}{2}{x}^{2}\sin \theta+{\gamma }_{x}\left(\dot{x}-\cos \theta \right)\right]{ds},$$

where $s$, ${s}_{1}$ $x$, and $\theta$ are the arc length, location of the rim, radial coordinate, and tilt angle, respectively (Supplementary Fig. 4a). ${\gamma }_{x}$ is the Lagrange multiplier for the geometric constraint $\dot{x}=\cos \theta$. The dots represented the derivative with respect to $s$. The membrane morphology at a given osmotic pressure difference $P$ was obtained by minimizing the free energy^46^. The difference in the concentration of particles, $c$, between the inside and outside of the IM was determined from the osmotic pressure difference using the van’t Hoff equation, $c=P/\left({N}_{A}{k}_{B}T\right)$ with the Avogadro number ${N}_{A}$.

The stable membrane morphology at several different particle concentration differences (i.e., osmotic pressure differences) is shown in Supplementary Fig. 4b. Here, the rim radius ${x}_{r}={\int }_{0}^{{s}_{1}}\cos \theta {ds}$ and total membrane area $A=4\pi {\int }_{0}^{{s}_{1}}{xds}$ were fixed to ${x}_{r}=12.5{nm}$ and $A=\pi \mu {m}^{2}$, respectively. As the concentration difference increased, the membrane adopted a more elongated shape.

### Statistics and reproducibility

All experiments were independently repeated at least two times with similar results. No statistical method was used to predetermine sample size. No data were excluded from the analyses. Cells for biochemical analysis and fields observed under the microscope are randomly selected. GraphPadPrism 8 Version 8.4.3 (GraphPad Software) was used to perform statistical tests.

### Reporting summary

Further information on research design is available in the Nature Portfolio Reporting Summary linked to this article.

### Supplementary information

Supplementary Information

Description of Additional Supplementary Files

Supplementary Movie 1

Supplementary Movie 2

Supplementary Movie 3

Supplementary Movie 4

Supplementary Movie 5

Reporting Summary

### Source data

Source Data

## Data Availability

The data supporting the finding of this study are available from the authors upon request. Source data for blots and graphs (Figs. 2c, d, 3c, d, 4, 5a–f, 6 and Supplementary Figs. 2a, b, d–h, 3) are provided as a Source Data file. Source data are provided with this paper.

## Code availability

ImageJ Scripts used in this study are provided in Supplementary Information.
