# Supplementary material for: A mechanism that ensures non-selective cytoplasm degradation by autophagy
Source: Nat Commun. 2023 Sep 19;14:5815. doi: 10.1038/s41467-023-41525-x (PMC10509180; doi:10.1038/s41467-023-41525-x)
Supplement: Supplementary file 1 — Supplementary Information [file 41467_2023_41525_MOESM1_ESM.pdf]

Supplementary Fig. 1

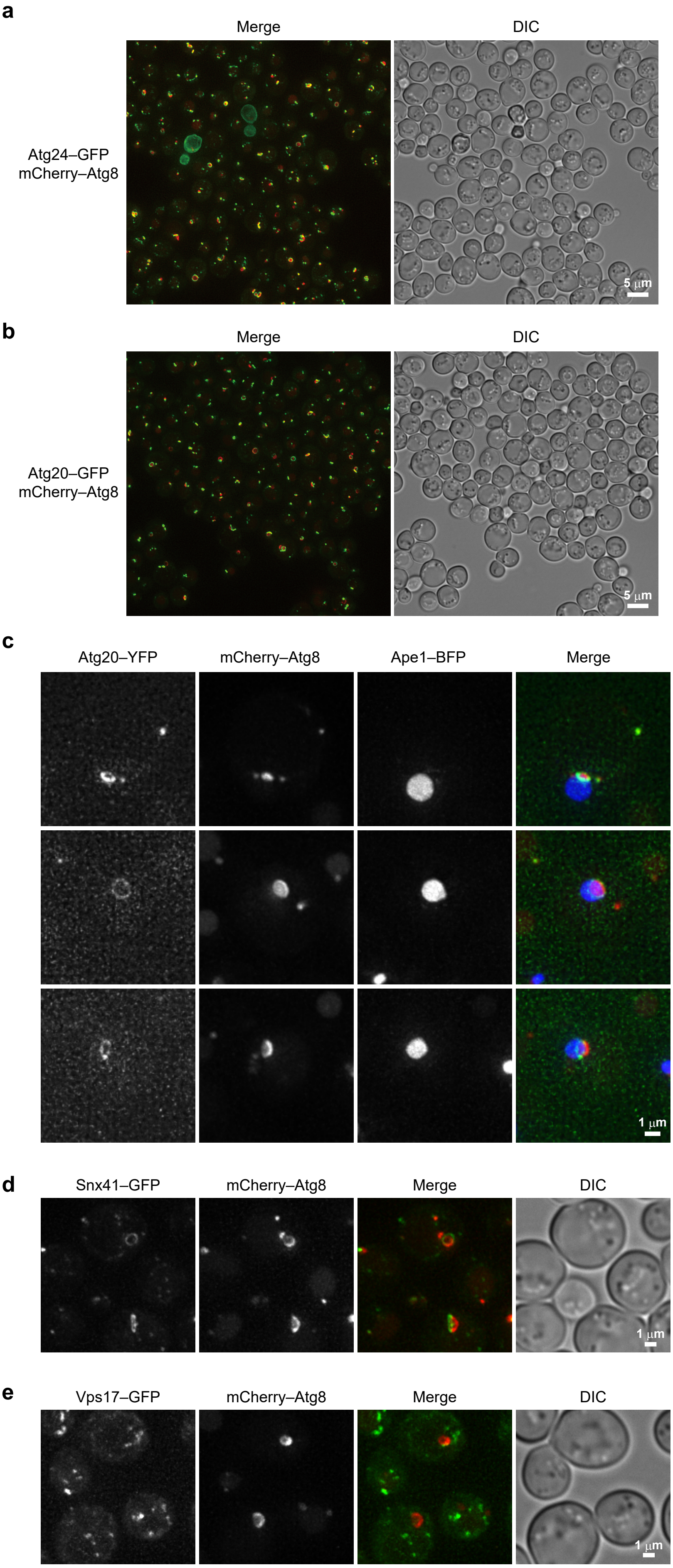

Supplementary Fig. 1. Supplemental data related to Fig. 1.

a, b, Zoom-out image of **Fig. 1a**, **c**. Scale bar, 5 μm. **c**, Ape1-overexpressing cells were treated with rapamycin for 4 h and observed under a fluorescence microscope. **d**, **e**, Ape1-overexpressing cells co-expressing mCherry–Atg8 and Snx41–GFP (**d**) or Vps17–GFP (**e**) were treated with rapamycin for 4 h and observed under a fluorescence microscope. Scale bar, 1 μm. All images except for the DIC image were subjected to maximum intensity projection.

Supplementary Fig. 2

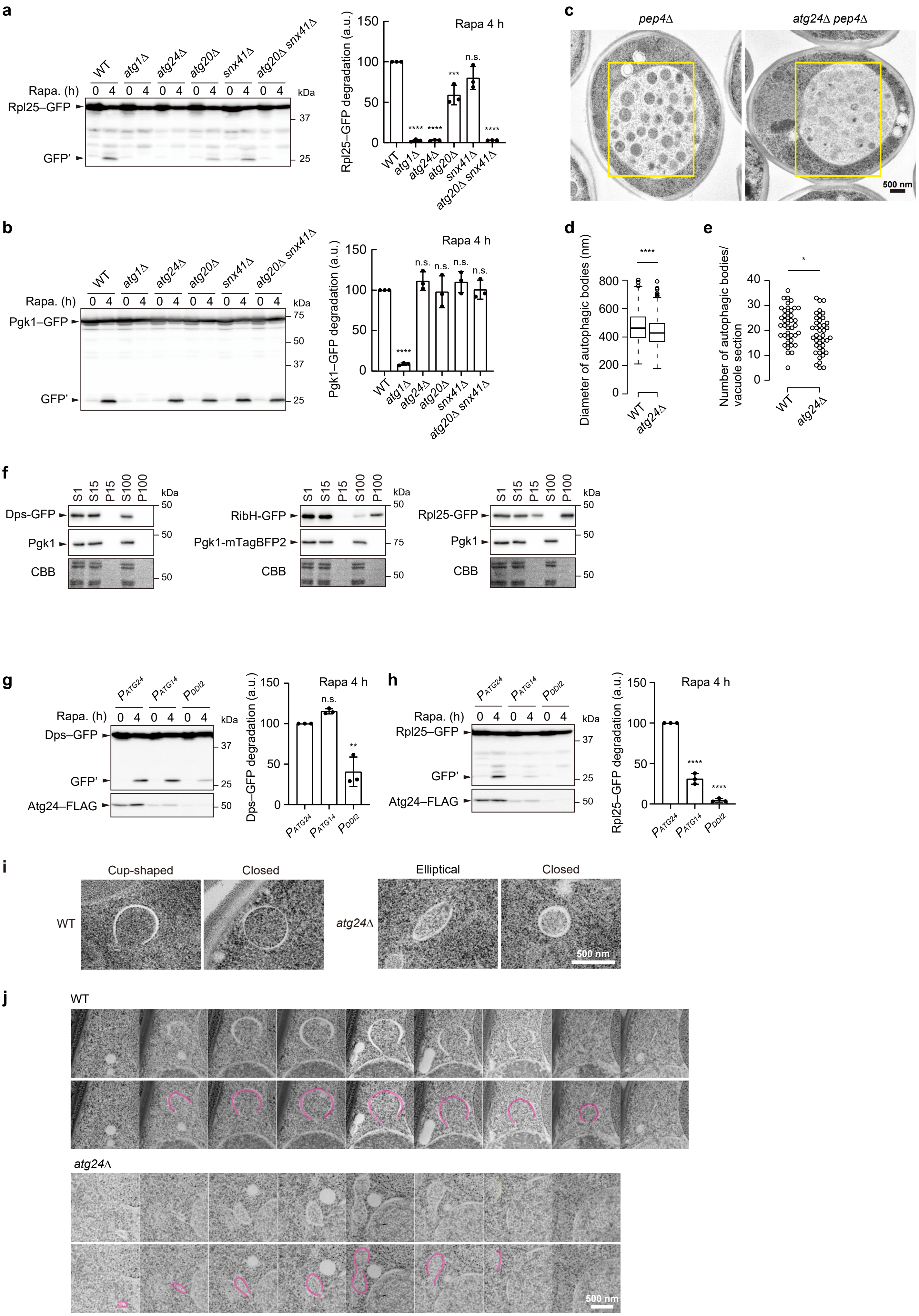

Supplementary Fig. 2. Supplemental data related to Fig. 2 and 3.

a, **b**, Yeast cells expressing Rpl25–GFP (**a**) or Pgk1–GFP (**b**) were treated with rapamycin and degradation of Rpl25–GFP (ribosomes) and Pgk1–GFP was examined by immunoblotting. Graphs show mean ± s.d. (n = 3) of ratio GFP′/(Rpl25–GFP or Pgk1–GFP + GFP′). ***, $P < 0.001$; ****, $P < 0.0001$; n.s., not significant (Tukey's multiple comparisons test, three independent experiments). **c**, Zoom-out image of **Fig. 2a**. **d**, **e**, In the experiment described in **Fig. 2a**, the cross-sectional diameter (**d**) and number (**e**) of autophagic bodies were measured and are shown as boxplots as defined in **Fig. 3c** and beeswarm plot, respectively. *, $P < 0.05$; ****, $P < 0.0001$ (Two-tailed Mann Whitney test, three independent experiments). (n = 909 (**d**, WT), 750 (**d**, *atg24*Δ), 41 (**e**, WT and *atg24*Δ)). **f**, Yeast cells were converted to spheroplasts, treated with rapamycin for 1 h, and then ruptured by filtration. Lysates were cleared by centrifugation at 1,000 g for 5 min (S1), and further centrifuged at 15,000 *g* for 30 min or at 100,000 *g* for 1 h to obtain pellets (P15 or P100) and supernatants (S15 or S100). The samples were analyzed by immunoblotting using antibodies against GFP and Pgk1 **g**, **h**, Yeast cells expressing Atg24–FLAG by the *ATG24* (P_{ATG24}), *ATG14* (P_{ATG14}) or *DDI2* (P_{DDI2}) promoter and expressing Dps–GFP (**g**) or Rpl25–GFP (**h**) were treated with rapamycin and degradation of Dps–GFP and Rpl25–GFP was examined by immunoblotting. Graphs show mean ± s.d. (n = 3) of ratio GFP′/(Dps–GFP or Rpl25–GFP + GFP′). **, $P < 0.01$; ****, $P < 0.0001$; n.s., not significant (Tukey's multiple comparisons test, three independent experiments). **i, j**, Cells were grown to mid-log phase in SD+CA+ATU and then incubated in SGly+CA+ATU for 2 h. Autophagosome-related structures in the cytosol were analyzed by single section (**i**) and serial section (**j**) electron microscopy. Scale bar, 500 nm. The IMs were traced with magenta lines (**j**). Source data and the exact adjusted *P*-values are provided as a Source Data file.

Supplementary Fig. 3

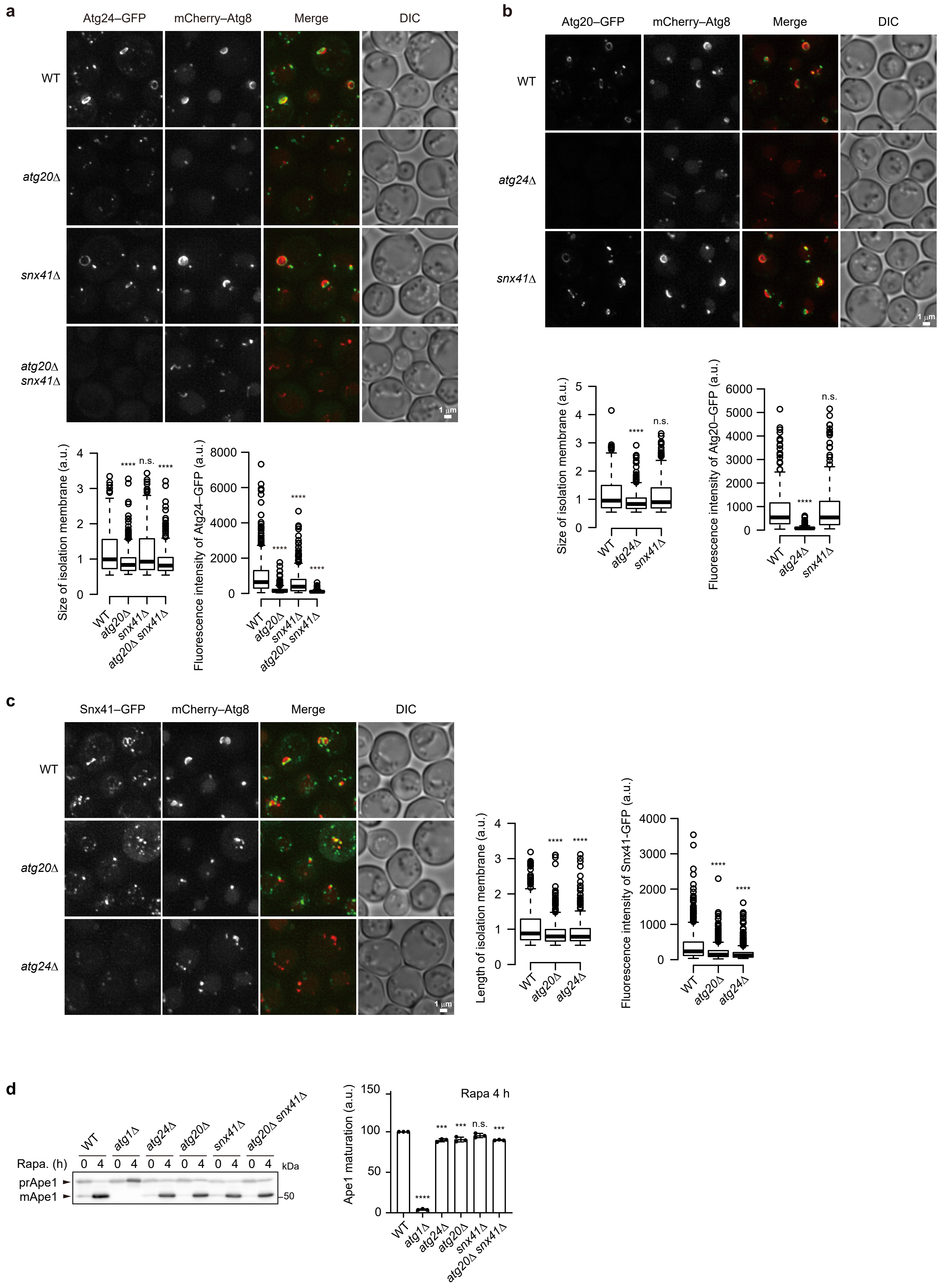

e

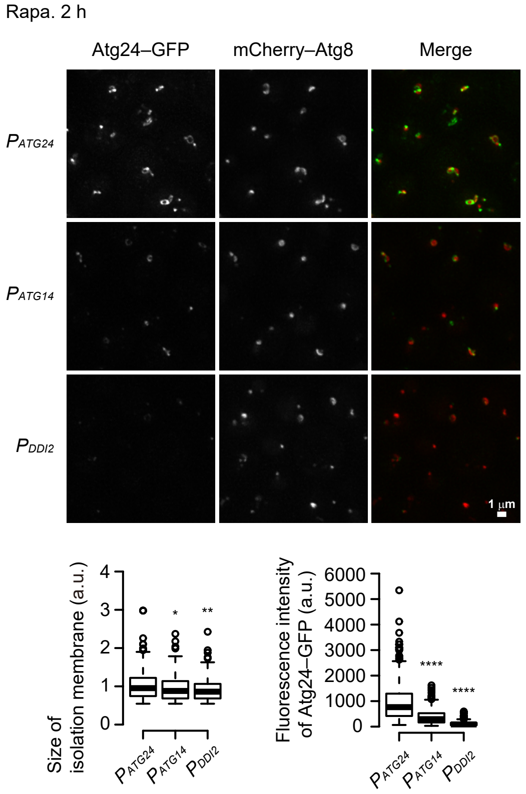
Rapa. 2 h
Atg24–GFP
mCherry–Atg8
Merge
P_{ATG24}
P_{ATG14}
P_{DDI2}
1 µm
Size of isolation membrane (a.u.)
Fluorescence intensity of Atg24–GFP (a.u.)
*
**
****
****
P_{ATG24} P_{ATG14} P_{DDI2}

f

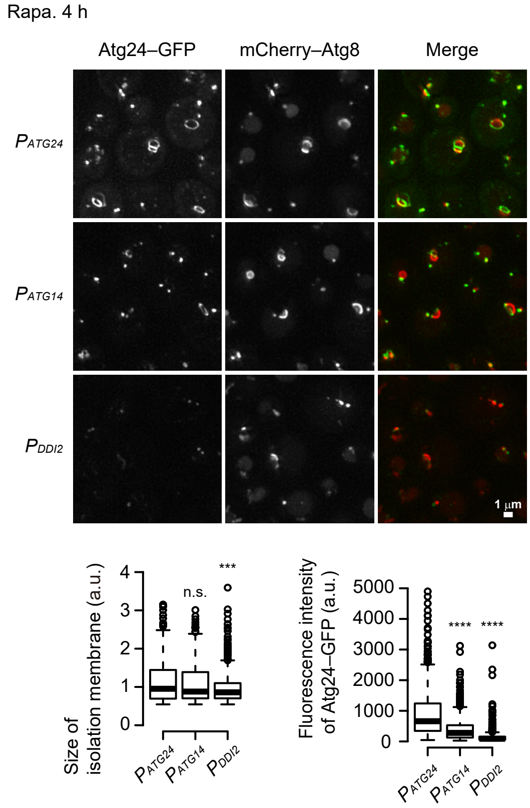
Rapa. 4 h
Atg24–GFP
mCherry–Atg8
Merge
P_{ATG24}
P_{ATG14}
P_{DDI2}
1 µm
Size of isolation membrane (a.u.)
Fluorescence intensity of Atg24–GFP (a.u.)
n.s.
***
****
****
P_{ATG24} P_{ATG14} P_{DDI2}

g

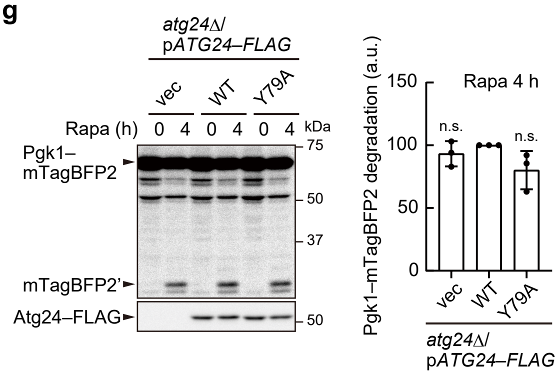
*atg24*Δ/ p*ATG24–FLAG*
vec WT Y79A
Rapa (h) 0 4 0 4 0 4
kDa
Pgk1–mTagBFP2
mTagBFP2'
Atg24–FLAG
75
50
37
50
Pgk1–mTagBFP2 degradation (a.u.)
Rapa 4 h
n.s.
n.s.
vec WT Y79A
*atg24*Δ/ p*ATG24–FLAG*

h

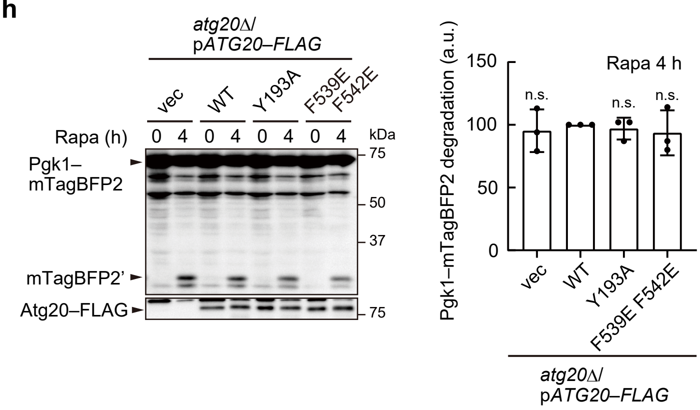
*atg20*Δ/ p*ATG20–FLAG*
vec WT Y193A F539E F542E
Rapa (h) 0 4 0 4 0 4 0 4
kDa
Pgk1–mTagBFP2
mTagBFP2'
Atg20–FLAG
75
50
37
75
Pgk1–mTagBFP2 degradation (a.u.)
Rapa 4 h
n.s.
n.s.
n.s.
vec WT Y193A F539E F542E
*atg20*Δ/ p*ATG20–FLAG*

Supplementary Fig. 3. Supplemental data related to Fig. 4 and 5.

a-c, e, f, Ape1-overexpressing cells were treated with rapamycin for 4 h (**a**-**c**, **f**) or 2 h (**e**) and observed under a fluorescence microscope. Scale bar, 1 μm. All images except for the DIC image were subjected to maximum intensity projection. The graphs show the sizes of IMs and fluorescence intensities of Atg24–GFP, Atg20–GFP and Snx41–GFP associated with IMs, measured as described in **Methods** and shown as boxplots as defined in **Fig. 3c**. *, $P < 0.05$; **, $P < 0.01$; ***, $P < 0.001$; ****, $P < 0.0001$; n.s., not significant (Dunn's multiple comparisons test, two independent experiments). (n = 405 (**a**, WT), 519 (**a**, *atg20*Δ), 409 (**a**, *snx41*Δ), 477 (**a**, *atg20*Δ *snx41*Δ), 314 (**b**, WT), 409 (**b**, *atg24*Δ), 400 (**b**, *snx41*Δ), 654 (**c**, WT), 673 (**c**, *atg20*Δ), 681 (**c**, *atg24*Δ), 283 (**e**, P_{ATG24}), 318 (**e**, P_{ATG14}), 257 (**e**, P_{DDI2}), 451 (**f**, P_{ATG24}), 392 (**f**, P_{ATG14}), 599 (**f**, P_{DDI2})). **d**, Yeast cells were grown to mid-log phase, treated with rapamycin, and examined by immunoblotting using anti-Ape1 antibodies. mApe1, mature Ape1; prApe1, Ape1 proform. Graphs show mean ± s.d. (n = 3) of ratio mApe1/(mApe1 + prApe1) at 4 h treatment of rapamycin. ***, $P < 0.001$; ****, $P < 0.0001$; n.s., not significant (Tukey's multiple comparisons test, three independent experiments). **g, h**, Atg24 or Atg20 mutant cells expressing Pgk1–mTagBFP2 were treated with rapamycin and examined by immunoblotting. Graphs show mean ± s.d. (n = 3) of ratio mTagBFP2′/(Pgk1–mTagBFP2 + mTagBFP2′). n.s., not significant (Tukey's multiple comparisons test, three independent experiments). Source data and the exact adjusted *P*-values are provided as a Source Data file.

Supplementary Fig. 4

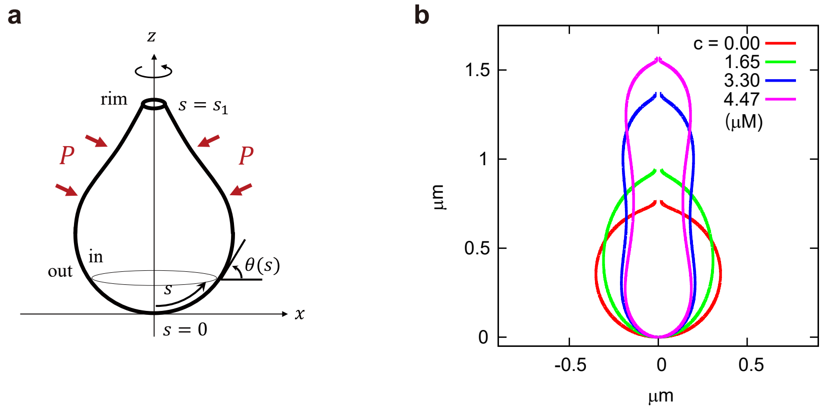

Supplementary Fig. 4. Model prediction of IM shapes.

a, A schematic description of model analysis of IM morphology. The inner and outer membranes are approximated by two paralleled axisymmetric membranes. z is the symmetric axis, s is the length along the contour measured from the origin, and θ is the angle between the tangent to the contour and the x-axis. P is the pressure applied to the membrane. The rim radius and membrane area were set to 12.5 nm and π μm^2, respectively. **b**, Membrane shapes were predicted by the bending energy model with different values of the osmotic pressure generated by the difference in concentration of particles between inside and outside the IM (see **Methods**). The predicted shapes of the IM at the concentration differences (c) of 0.00 (red), 1.65 (green), 3.30 (blue) and 4.47 μM (purple) are shown.

Table S1

Yeast strains used in this study

Name	Genotype	Figures
W303-1A	*MAT*a *ade2-1 ura3-1 his3-11,15 trp1-1 leu2-3,112 can1-100*	(Thomas and Rothstein, 1989)
ScTK1044	W303-1A *ade2*::*ADE2 APE1-mTagBFP2-kanMX4 his3*::pRS303-P_{GPD}-*APE1 leu2*::*mCherry-ATG8*$^{\Delta R117}$-*zeoNT3*	4
ScTK1186	ScTK1044 *ATG20–EGFP-hphNT1*	1c, S1b, S3b, SM1
ScTK1187	ScTK1044 *ATG24–EGFP-hphNT1*	1a, S1a, S3a, S3e, f, SM1
ScTK1188	ScTK1044 *SNX41–EGFP-hphNT1*	S1d, S3c
ScTK1189	ScTK1044 *VPS17–EGFP-hphNT1*	S1e
ScTK1190	ScTK1044 *ATG24–sYFP-hphNT1*	1b
ScTK1374	ScTK1044 *ATG20–sYFP-hphNT1*	S1c
YNH1577	W303-1A, *ade2*::*ADE2 PGK1–EGFP-kanMX4*	S2b
YNH1584	YNH1577 *atg1*Δ::*zeoNT3*	S2b
ScTK1339	YNH1577 *atg24*Δ::*hphNT1*	S2b
ScTK1340	YNH1577 *atg20*Δ::*hphNT1*	S2b
ScTK1341	YNH1577 *snx41*Δ::*hphNT1*	S2b
ScTK1342	ScTK1340 *snx41Δ*::*natNT2*	S2b
ScTK1429	YNH1577 *pep4*Δ::*natNT2*	2a, S2c, S2d, e, SM2
ScTK1430	YNH1577 *atg24*Δ::*hphNT1 pep4*Δ::*natNT2*	2a, S2c, S2d, e, SM2
ScTK1343	W303-1A, *ade2*::*ADE2* P_{CYC1}-*Dps(Opti)–EGFP*_pRS303	2c, S2f, SM3
ScTK1344	ScTK1343 *atg1*Δ::*hphNT1*	2c
ScTK1345	ScTK1343 *atg24*Δ::*hphNT1*	2c
ScTK1346	ScTK1343 *atg20*Δ::*hphNT1*	2c
ScTK1347	ScTK1343 *snx41*Δ::*hphNT1*	2c
ScTK1348	ScTK1346 *snx41*Δ::*natNT2*	2c
ScTK1504	ScTK1343 *ATG24–3×FLAG-hphNT1*	S2g
ScTK1506	ScTK1343 *natNT2*-P_{ATG14}-*ATG24–3×FLAG-hphNT1*	S2g
ScTK1505	ScTK1343 *natNT2*-P_{DDI2}-*ATG24–3×FLAG-hphNT1*	S2g
ScTK1350	W303-1A, *ade2*::*ADE2* P_{CYC1}-*RibH(Opti)–EGFP_pRS303 PGK1–mTagBFP2-KanMX4*	2d, S2f, SM3
ScTK1351	ScTK1350 *atg1*Δ::*hphNT1*	2d
ScTK1352	ScTK1350 *atg24*Δ::*hphNT1*	2d, 5e, S3g
ScTK1353	ScTK1350 *atg20*Δ::*hphNT1*	2d, 5f, S3h
ScTK1354	ScTK1350 *snx41*Δ::*hphNT1*	2d
ScTK1377	ScTK1353 *snx41*Δ::*natNT2*	2d
ScTK783	W303-1A, *ade2*::*ADE2 leu2*::*mCherry–ATG8*$^{\Delta R117}$-*zeoNT3*	-
ScTK1848	ScTK783 *ATG24–mNeonGreen-hphNT1*	3c, d
ScTK1867	ScTK1848 *atg20*Δ::*kanMX*	3c
ScTK1868	ScTK1848 *snx41*Δ::*kanMX*	3c
ScTK1873	ScTK1868 *atg20*Δ::*natNT2*	3c

ScTK1859	ScTK783 *natNT2-P$_{DDI2}$-ATG24–mNeonGreen-hphNT1*	3d
ScTK1860	ScTK783 *natNT2-P$_{ATG14}$-ATG24–mNeonGreen-hphNT1*	3d
ScTK1398	ScTK1044 *atg2Δ::hphNT1*	4
ScTK1046	ScTK1044 *atg20Δ::hphNT1*	4
ScTK1047	ScTK1044 *atg24Δ::hphNT1*	4
ScTK1048	ScTK1044 *snx41Δ::hphNT1*	4
ScTK1399	ScTK1046 *snx41Δ::natNT2*	4
ScTK1234	ScTK1046 *ura3*::pRS306-*ATG20–EGFP*	5b
ScTK1235	ScTK1046 *ura3*::pRS306-*atg20^{Y193A}–EGFP*	5b
ScTK1236	ScTK1046 *ura3*::pRS306-*atg20$^{F539E\ F542E}$–EGFP*	5b
ScTK1238	ScTK1047 *ura3*::pRS306-*ATG24–EGFP*	5a
ScTK1239	ScTK1047 *ura3*::pRS306-*atg24^{Y79A}–EGFP*	5a
ScTK1407	W303-1A, *ade2::ADE2* his3::pRS303-*RPL25–EGFP*	S2a, S2f
ScTK1419	ScTK1407 *atg1Δ::hphNT1*	S2a
ScTK1421	ScTK1407 *atg24Δ::hphNT1*	5c, S2a
ScTK1422	ScTK1407 *atg20Δ::hphNT1*	5c, S2a
ScTK1423	ScTK1407 *snx41Δ::hphNT1*	S2a
ScTK1428	ScTK1422 *snx41Δ::natNT2*	S2a
ScTK1507	ScTK1407 *ATG24–3×FLAG-hphNT1*	S2h
ScTK1509	ScTK1407 *natNT2-P$_{ATG14}$-ATG24–3×FLAG-hphNT1*	S2h
ScTK1508	ScTK1407 *natNT2-P$_{DDI2}$-ATG24–3×FLAG-hphNT1*	S2h
YNH1577	W303-1A, *ade2::ADE2*	6, S3d
ScTK1415	YNH1577 *atg1Δ::hphNT1*	6, S3d
ScTK1416	YNH1577 *atg11Δ::hphNT1*	6
ScTK1405	YNH1577 *atg24Δ::hphNT1*	6, S3d
ScTK1417	YNH1577 *atg20Δ::hphNT1*	6, S3d
ScTK1418	YNH1577 *snx41Δ::hphNT1*	6, S3d
ScTK1427	ScTK1417 *snx41Δ::natNT2*	6, S3d
ScTK479-1	W303-1A *ade2::ADE2 leu2::GFP–ATG8-hphNT1 his3*::pRS303-*ATG8*	3a, S2i-j
ScTK1049-2	ScTK479-1 *atg24Δ::zeoNT3*	3a, S2i-j
ScTK1057-2	ScTK1049-2 *atg1Δ::kanMX4*	3a
ScTK2009	ScTK783 *atg24Δ:: kanMX4 ATG1–EGFP-natNT2*	3b
ScTK1369	ScTK1187 *atg20Δ::natNT2*	S3a
ScTK1370	ScTK1187 *snx41Δ::natNT2*	S3a
ScTK1371	ScTK1369 *snx41Δ::CgTRP1*	S3a
ScTK1367	ScTK1186 *atg24Δ::natNT2*	S3b
ScTK1368	ScTK1186 *snx41Δ::natNT2*	S3b
ScTK1372	ScTK1188 *atg24Δ::natNT2*	S3c
ScTK1373	ScTK1188 *atg20Δ::natNT2*	S3c
ScTK1466	ScTK1044 *natNT2-P$_{ATG14}$-ATG24–EGFP-hphNT1*	S3e, f
ScTK1483	ScTK1044 *natNT2-P$_{DDI2}$-ATG24–EGFP-hphNT1*	S3e, f
ScTK2004	ScTK479-1 *pep4Δ::kanMX4 VPH1-2×mCherry-natNT2*	SM4
ScTK2005	ScTK1049-2 *pep4Δ::kanMX4 VPH1-2×mCherry-natNT2*	SM5

Table S2

Plasmids used in this study

Name	Description	Figures
pTKO402	pRS316-*ATG24-FLAG*	5c, e, S3g
pTKO406	pRS316-*atg24*^{Y79A}-*FLAG*	5c, e, S3g
pTKO407	pRS316-*ATG20-FLAG*	5d, f, S3h
pTKO408	pRS316-*atg20*^{Y193A}-*FLAG*	5d, f, S3h
pTKO409	pRS316-*atg20*$^{F539E\ F542E}$-*FLAG*	5d, f, S3h

ImageJ Script S1

```
run("Set Measurements...", "perimeter redirect=None decimal=9");

rename("Atg8");

run("Smooth");

run("Sharpen");


//maximum intensity projection

run("Z Project...", "projection=[Max Intensity]");

run("Split Channels");


//extract Ape1 complex

selectWindow("C2-MAX_Atg8");

setAutoThreshold("Otsu dark");

setOption("BlackBackground", true);

run("Convert to Mask");

run("Maximum...", "radius=2");


//find the isolation membranes

selectWindow("C1-MAX_Atg8");
```

```
setAutoThreshold("Moments dark");

run("Convert to Mask");

imageCalculator("Multiply create", "C1-MAX_Atg8","C2-MAX_Atg8");

selectWindow("Result of C1-MAX_Atg8");

run("Analyze Particles...", "size=0.10-Infinity exclude add in_situ");



//Measure the perimeter of the isolation membrane

roiManager("Measure");

roiManager("Deselect");

roiManager("Delete");

run("Close All");
```

ImageJ Script S2

```
run("Set Measurements...", "area mean perimeter redirect=None decimal=9");

rename("Atg8");

run("Duplicate...", "duplicate");

selectWindow("Atg8");

run("Smooth");

run("Sharpen");



//maximum intensity projection

run("Z Project...", "projection=[Max Intensity]");

run("Split Channels");

close();

selectWindow("C1-MAX_Atg8");



//find the isolation membranes

setAutoThreshold("Moments dark");

run("Convert to Mask");

run("Analyze Particles...", "size=0.10-Infinity exclude add in_situ");

close("C1-MAX_Atg8");
```

```
close("C2-MAX_Atg8");

selectWindow("Atg8-1");

run("Z Project...", "projection=[Max Intensity]");

run("Split Channels");

selectWindow("C2-MAX_Atg8-1");


//Measure the perimeter of the isolation membrane

roiManager("Measure");

roiManager("Deselect");

roiManager("Delete");

run("Close All");
```

ImageJ Script S3

```
run("Set Measurements...", "fit redirect=None decimal=9");

rename("IM");



//maximum intensity projection

run("Z Project...", "projection=[Max Intensity]");

run("Split Channels");

selectWindow("C2-MAX_IM");

run("Smooth");

run("Smooth");

run("Smooth");

selectWindow("C1-MAX_IM");

run("Smooth");

run("Smooth");

run("Smooth");



//find the opening of the isolation membranes

setThreshold(800, 65535);

setOption("BlackBackground", false);
```

```
run("Convert to Mask");

run("Divide...", "value=255");

run("Maximum...", "radius=5");

imageCalculator("Multiply create", "C2-MAX_IM","C1-MAX_IM");


//Measure the opening of the isolation membrane

selectWindow("Result of C2-MAX_IM");

setAutoThreshold("Default dark");

setOption("BlackBackground", false);

run("Convert to Mask");

run("Analyze Particles...", "size=0-2 display exclude include add in_situ");

run("Close All");

roiManager("Deselect");

roiManager("Delete");
```
