# Supplementary material for: A mechanism that ensures non-selective cytoplasm degradation by autophagy
Source: Nat Commun. 2023 Sep 19;14:5815. doi: 10.1038/s41467-023-41525-x (PMC10509180; doi:10.1038/s41467-023-41525-x)
Supplement: Supplementary file 2 — Description of Additional Supplementary Files [file 41467_2023_41525_MOESM2_ESM.pdf]

Description of additional supplementary files

Supplementary Movie 1

Z-stack image sequence of Atg24–GFP and Atg20–GFP shown in Fig. 1a and c.

Supplementary Movie 2

Observation of Pgk1–GFP sequestered into autophagic bodies in yeast cells. *pep4*Δ cells expressing Pgk1–GFP were grown to mid-log phase, treated with rapamycin for 24 h and examined by fluorescence microscopy. Time-lapse images were obtained at 32.65 ms per frame.

Supplementary Movie 3

Observation of Dps–GFP and RibH–GFP in yeast cells. Cells expressing Dps–GFP or RibH– GFP were grown to mid-log phase and observed by fluorescence microscopy. Time-lapse images were obtained at 32.65 ms per frame.

Supplementary Movie 4 and 5

Wild-type (Video 4) and *atg24*Δ (Video 5) cells expressing GFP–Atg8 and Vph1–2×mCherry were treated with rapamycin. Fluorescence images were taken at 1 min intervals soon after rapamycin addition.
